# Healthcare Databases in Thailand and Japan: Potential Sources for Health Technology Assessment Research

**DOI:** 10.1371/journal.pone.0141993

**Published:** 2015-11-11

**Authors:** Surasak Saokaew, Takashi Sugimoto, Isao Kamae, Chayanin Pratoomsoot, Nathorn Chaiyakunapruk

**Affiliations:** 1 Center of Health Outcomes Research and Therapeutic Safety (Cohorts), School of Pharmaceutical Sciences, University of Phayao, Phayao, Thailand; 2 The Canon Institute for Global Studies, Tokyo, Japan; 3 Graduate School of Public Policy, University of Tokyo, Tokyo, Japan; 4 Meiji Institute for Global Affairs, Meiji University, Tokyo, Japan; 5 Center of Pharmaceutical Outcomes Research (CPOR), Faculty of Pharmaceutical Sciences, Naresuan University, Phitsanulok, Thailand; 6 Faculty of Public Health, Naresuan University, Phitsanulok, Thailand; 7 School of Pharmacy, Monash University Sunway Campus, Selangor, Malaysia; 8 School of Population Health, University of Queensland, Brisbane, Australia; 9 School of Pharmacy, University of Wisconsin, Madison, United States of America; UNAIDS, TRINIDAD AND TOBAGO

## Abstract

**Background:**

Health technology assessment (HTA) has been continuously used for value-based healthcare decisions over the last decade. Healthcare databases represent an important source of information for HTA, which has seen a surge in use in Western countries. Although HTA agencies have been established in Asia-Pacific region, application and understanding of healthcare databases for HTA is rather limited. Thus, we reviewed existing databases to assess their potential for HTA in Thailand where HTA has been used officially and Japan where HTA is going to be officially introduced.

**Method:**

Existing healthcare databases in Thailand and Japan were compiled and reviewed. Databases’ characteristics e.g. name of database, host, scope/objective, time/sample size, design, data collection method, population/sample, and variables were described. Databases were assessed for its potential HTA use in terms of safety/efficacy/effectiveness, social/ethical, organization/professional, economic, and epidemiological domains. Request route for each database was also provided.

**Results:**

Forty databases– 20 from Thailand and 20 from Japan—were included. These comprised of national censuses, surveys, registries, administrative data, and claimed databases. All databases were potentially used for epidemiological studies. In addition, data on mortality, morbidity, disability, adverse events, quality of life, service/technology utilization, length of stay, and economics were also found in some databases. However, access to patient-level data was limited since information about the databases was not available on public sources.

**Conclusion:**

Our findings have shown that existing databases provided valuable information for HTA research with limitation on accessibility. Mutual dialogue on healthcare database development and usage for HTA among Asia-Pacific region is needed.

## Introduction

Health technology assessment (HTA) has been defined as “A multidisciplinary field of policy analysis that studies the medical, social, ethical, and economic consequences of health-care interventions”[1]. It is a process for evaluation of new healthcare intervention that examines the available information to aid decision making. Over the last decade, the global use of HTA for value-based healthcare decisions has seen a continuous growth [2–4]. Notably in the Asian Region, South Korea, Taiwan and Thailand had formally adopted HTA as part of their decision making on health policy [5–7]. The adoption of HTA in these three countries was driven by a number of factors mainly a clear direction towards universal healthcare coverage and the need for rational allocation of scarce resources.

It is well-recognized that HTA is a dynamic process as such conducting one requires timely supply of research findings to inform decisions about the value of the interventions in question. Hence, information in form of healthcare databases is considered as an essential part of HTA research. These databases had become a widely accepted source of information, especially in the Western countries. To date, there has been a number of HTA performed that utilized healthcare databases to produce meaningful and contextually relevant findings for policy makers. For examples, in the United States (US), administrative claim data were used by patient outcome research teams (PORT) to assess the effectiveness of a number of treatments [8]. Denmark had developed standards for use of its Biobank, long-term storage of biological samples from patients used in studies of disease control and treatment, which included personal identifiers [9]. Sweden has over fifty quality registries, which comprised of patient data, diagnoses, interventions, and outcomes [10]. Previous reports have made known repositories of available databases in the US and Canada [11,12], which consequently facilitated the use of databases.

In contrast, the number of HTA research which makes use of healthcare databases in Asia-Pacific countries is relatively small, despite the availability of such databases. It is due to the well-recognized fact that the accessibility to these databases has proven to be difficult [13]. Furthermore, there is a lack of formal repositories. Thus, having a repository of existing healthcare databases made known to the public may be regarded as the first step to accessibility, consequently leads to an increase of use, and in turn the number of HTA research in Asia-Pacific region.

This study aimed to compile and describe healthcare databases currently available in Thailand and Japan, where their HTA use is at different stages. Thailand is an example of a country with a long history of HTA development [5]. The economic recession and health financing reforms played a crucial role in driving the demands for HTA research in aiding policy makers. At a different stage of HTA adoption, Japan is another country with a long history of interest in using HTA but has minimal evidence to demonstrate the use of HTA in policy decision making [7,14]. In 2012, the Japanese Government has demonstrated a strong interest in adopting the use of HTA in their policy decision making process. The formal adoption of HTA in Japan is expected to be introduced in the near future [15]. It is believed that the compilation as well as the assessment of variables of existing healthcare databases, particularly in terms of potential use for HTA research, would encourage further research development in HTA, not only in Thailand and Japan but also the Asia-Pacific region.

## Methods

Since there are no comprehensive lists of healthcare databases available in Thailand and Japan, we compiled the lists through a combination of search engines (e.g. Google, and PubMed), references from published articles, and a network of people with experience in healthcare databases through February 2013 without language restriction. Included databases’ characteristics were listed and described e.g. name of database, host, scope/objective, time/sample size, design, data collection method, population/sample, and variables. Then, databases were classified into three groups according to sources and methods of data collection including: 1) population and household surveys, 2) surveillances and registries, and 3) administrative and claim databases.

To be included in our compiled list of databases, such databases must be of value for potential use in HTA. That is databases must at least identify one of the following domains according to *‘best practice in undertaking and reporting health technology assessment’* [16] including; safety/efficacy/effectiveness, social/ethical, organization/professional, economic, and epidemiological domains. The details of outcomes/variables in each domain are available in Table 1. Request route for the access of each database was also provided.

**Table 1:** Domains of databases with potential for use in HTA*.

Domain	Outcomes/variables
Safety/Efficacy/ Effectiveness	Mortality
	Morbidity
	Disability
	Adverse event
	Quality of life (e.g. EQ-5D, SF36, HUI)
	Life year, Quality-adjusted life year
Social/ethical	Compliance
	Acceptance
	Satisfaction
Organizational/professional	Utilization (e.g. service or technology utilization)
	Length of stay
	Personnel required
	Material required (e.g. hospital bed)
Economic	Cost/price
	Income/economic status
Epidemiological	Prevalence
	Incidence
	Health state
	Demographic (e.g. age, sex, education)

Adapted from Draborg *et al* [1] and Velasco *et al*.2002 [16]

* **HTA**, Health technology assessment; **EQ-5D**, European Quality of Life-5 Dimensions; **SF36**, Medical Outcomes Study 36-Item Short-Form Health Survey; **HUI**, Health Utility Index

## Results

We included 40 databases, 20 from Thailand and 20 from Japan, as a representation of available databases. Our compilation of databases revealed that most data were of national representativeness. Sixty percent (24 of 40) were longitudinal data. These databases serve as foundations for the research in health technology assessment and evaluation for health decision making. Host agencies take primary responsibility for designing, collecting, maintaining and updating datasets, as well as disseminating the data. Characteristics of databases were listed and described in terms of name of database, host, scope/objective, time/sample size, design, data collection method, population/sample, and variables as shown in Table 2. These 40 databases from Thailand and Japan, were classified into three groups; 1) population and household surveys, 2) surveillances and registries, and 3) administrative and claim databases (Table 3).

**Table 2:** Characteristics of databases.

Database		Host	Scope/objective	Time and size	Design	Data collection method	Population/sample	Variables/health-related parameters
	**Thailand**							
1	Population and Housing Census (PHC)	NSO	Collect basic information on the number of population on demographic and socioeconomic characteristics as well as housing characteristics of everyone who residing in the country.	Every 10 y (1909–2010); (n = 20.3 million households)	Cross-sectional	Face to face interview; Self-enumeration; Internet (http://popcensus.nso.go.th); Telephone Interview Center	All residents in all provinces, and also in both municipal and non-municipal areas throughout the country (excluding households of foreign diplomats and other temporary residents, non-Thai who live in Thailand less than three months prior to the Census Day, refugees or illegal migrants)	Demographic data: age, sex, religion, nationality, speaking languages, type and characteristics of household; Education: number of students currently enrolled in each level, number of graduates; Employment: type of work and work status such as major occupation and industry; Fertility: marital status, number of children ever born, number living and deceased children; Migration: place of birth, duration of living in current place and reason for migration; Housing: type and characteristics of dwelling, tenure of dwelling, use of light and toilet facility, type of fuel, drinking water and water supplies, ownership of basic living appliances and use of different types of technology.
2	Health and Welfare Survey (HWS)	NSO	Collect information on the health insurance, illness, health services, payment, equity, injury, and co-morbidity	Annual (1974–1978); Every 5 y (1981–2001); Annual (2003 onward); (n = 26,000 households)	Cross-sectional	Interview	All private, non-institution households residing in all provinces, and also in both municipal and non-municipal areas	Demographic, geographic, education, occupation; Health insurance scheme, disease, illness, health services, accident and injury, DM, HTN, breast cancer screening, cervical cancer screening, dental health; Out-of-pocket payment
3	Socio-Economic Survey (SES)	NSO	Survey information on income, expenditure, debt, and income distribution of household.	Every 5 y (1957–1986); Every 2 y (1987–2004); Annual (2006 onward); (n = 52,000 households)	Cross-sectional	Interview	All private, non-institution households residing in all provinces, and also in both municipal and non-municipal areas (excluding households of foreign diplomats and other temporary residents)	**Household members and expenditures:** Household membership; Housing characteristics; Expenditure on goods and services; Expenditure on food, beverages and tobacco, **Household members and expenditures:** Income from wages and salaries; Income from business, industry, or profession other than farming; Income from farm business; Income from other sources; Household asset and debt; Migration and remittance transfer; Demographic, geographic, education, occupation
4	Reproductive Health Survey (RHS)	NSO	Survey on family planning, maternal and child health, AIDS, breast and cervical cancer, infertility, sex education, and adolescent health.	1975, 1985, 1996, 2006; 2009 (n = 37,511 women, and 11,971 adolescents)	Cross-sectional	Interview	Women age 15–59, adolescent age 15–24 (both men and women) throughout the country	Family planning: Pre-marital preparation (counseling, HIV, thalassemia screening), age at first marriage, number of children born alive; Maternal and Child Health: Child delivery, breastfeeding; Screening for breast and cervical cancer; Demographic, geographic, education, occupation
**5**	National Disability Survey (NDS)	NSO	Survey of disabilities on health care	2002, 2007; Every 5 years (2007 onward); (n = 1.9 million disabilities)	Cross-sectional	Interview	All private, non-institution households residing in all provinces, and also in both municipal and non-municipal areas (excluding households of foreign diplomats and other temporary residents)	Disability, impairments, activity limitations, participation restriction, health-related problems, devices used, medications, demographic, geographic, education, occupation
**6**	Multiple Indicator Cluster Survey(MICS)	NSO	Monitor children and women status	2005–2006; (n = 43,440)	Cross-sectional	Interview using questionnaires for: Households, Women 15–49 y, Children <5 y	Children (< 5 y) and women (15–49 y) throughout the country	Demographic, geographic, education, occupation; Nutritional status, breastfeeding, salt iodization, low birth weight, immunization, water and sanitation, slum household, contraception, maternal and newborn health, child development, literacy, early marriage, disability, HIV/AIDS knowledge and attitude, support to orphaned and vulnerable children
7	National Health Examination Survey(NHES)	HSRI	Survey the prevalence and risk factors of health-related problems	1^st^: Aug 1, 1991 –Mar 31, 1992; (n = 22,217)	Cross-sectional	Interview; Physical examination; Laboratory tests in 17 provinces	0–5 years	Height, weight, development; Interview: family profiles, income, general information
							6–14 years	Height, weight; Interview: family profiles, income, general information
							15–29 years	Height, weight, physical exam, disability, peak flow rate, liver function, CBC, Hct, serum protein, serum creatinine, FBS, lipid profile, total bilirubin,
							≥ 30 years	Height, weight, breast test (women), disability, peak flow rate, liver function, CBC, Hct, serum protein, serum creatinine, FBS, lipid profile, total bilirubin, EKG, chest X-ray, plain KUB
							≥15 years	Interview: family profiles, income, general information, smoking, alcohol, illness, injury, history (seizure, cirrhosis, stone, pain, cervical cancer screening (women), DM, HTN, Dyslipidemia, asthma, COPD, TB, allergy, angina pectoris
				2^nd^: Jun–Oct 1997 (n = 16,182)	Cross-sectional	Interview; Physical examination; Laboratory tests; in 9 provinces	≤ 6 years	Development and related-factors
							6–12 years	Intelligence and related-factors (TONI 2)
							13–59 years	Height, weight, BMI, BP, waist circumference, hip circumference, eye exam, Hb, Hct, FBS, total cholesterol; Interview: general information, health behavior, smoking, alcohol
							≥ 60 years	Dependency, disability, dementia,
				3^rd^: 2003–2004; (n = 39,290)	Cross-sectional	Interview; Physical examination; Laboratory tests in 37 provinces	15–59 years	Height, weight, BMI, BP, waist circumference, hip circumference, eye exam, Hb, Hct, FBS, total cholesterol; Interview: general information, health behavior, smoking, alcohol, underlying disease, illness, injury, sexual behavior, medications history
							≥ 60 years	Same as 15–59 years plus health status, daily activities, dementia, temporomandibular disorders, income, health insurance and welfare
				4^th^: 2008–2009 (n = 20,450)	Cross-sectional	Interview; Physical examination; Laboratory tests in 21 provinces	≥ 15 years	Height, weight, BMI, BP, waist circumference, hip circumference, eye exam, Hb, Hct, FBS, total cholesterol; Interview: general information, health behavior, smoking, alcohol, underlying disease, illness, injury, sexual behavior, medications history
8	National Epidemiology Survey on Mental Health (NESMH)	DMH	Survey of prevalence of mental-related health problem	1998, 2003 (n = 11,685)	Cross-sectional	Questionnaire; AUDIT screening test; Mini International Neuropsychiatric interview	15–59 y men and women throughout the country (after sampling: 36 provinces + BKK)	Demographic, geographic, education, occupation, asset, income, suicidal risk, major depressive episode, generalized anxiety disorder, dysthymia, agoraphobia, psychotic disorder, hypomanic episode, mood disorder with psychotic features, manic episode, panic disorder
9	National Nutrition Survey (NNS)	BN	Survey of food status, nutrition, and behavior	1960, 1975, 1986, 1995, 2003 (n = 4,083 household, 19,956 persons)	Cross-sectional	Interview; Anthropometry; Physical examination; Laboratory	All age throughout the country (after sampling: 10 provinces)	Demographic, geographic, education, occupation, income, expenditure, nutritional status, food, sanitation, clinical assessment and anthropometric measurement, iodine deficiency, jaundice, Hb, Hct, glucose, TC, TG, HDL, LDL, Vit. B1 deficiency, Vit. A deficiency, food consumption behavior
10	Behavioral Risk Factors Surveillance System (BRFSS)	BNCD	Survey aimed to establish the data base system of health behaviors (non-communicable diseases and injuries) of the population	2003, 2004, 2005, 2006 (n = 65,542, included 32,518 males and 33,024 females)	Cross-sectional	Interview; Questionnaires	The individuals of Thai citizenship aged 15–74 years, who resided at home, excluding institutions, such as dormitory, military camp, and so on (after sampling: 37 provinces + BKK)	Demographics (sex, age, place of birth, religion, marriage status, education, employment status, work status, income, weight, height and waist circumference), general health status, accessibility to health services, HTN, DM, chronic diseases, physical activity, fruit and vegetable consumption, road traffic injuries, tobacco consumption, alcohol consumption, cervical cancer examination, HIV/AIDS examination, knowledge of selected NCD prevention
11	Cancer Registry (CR)	NCI	To study the incidence, prevalence, diagnostic method, type of cancer, cancer stage, treatment, and outcome	1986–2007 (n = 241,051 included 121,986 males and 119,065 females during 2001–2003)	Longitudinal	Registry (case report, ultrasound, pathological exam). All registered cases are followed up by passive and active procedures. Registered cases are matched with death certificates. For the remaining cases, follow up information was obtained by scrutiny of hospital records, postal enquiries, and through the network of health care system.	All ages in 12 provinces + BKK	Demographic, geographic, registry number, residential address, date of birth, age, sex, date and method of diagnosis, topographic site, histology and extent of cancer, and vital status of cancer patient, type of CA cases, test finding, treatment, death, cause of death. The data collection in details may differ from one to another registry.
12	Thai Diabetes Registry (TDR)	TES	To identify the characteristics of Thai diabetic patients in tertiary care medical center sand to determine the extent of long term diabetic complications	Apr 2003-Feb 2006 (n = 9,419)	Longitudinal	Registry (case report, interview, anthropometry, physical examination, laboratory)	11 participating medical centers	Demographic data, health insurance, type of DM, alcohol consumption, cigarette smoking, specific medications (including insulin, oral hypoglycemic agents, antihypertensive agents, lipid-lowering agents and aspirin), pertinent parts of physical examinations, laboratory examinations performed, diabetic complications verified by physicians’ reports, fasting serum glucose, serum total cholesterol, HDL cholesterol (HDL-C) and triglyceride levels, glycosylated hemoglobin (HbA1c), and serum creatinine, LDL cholesterol (LDL-C), BP, health insurance, death from cardiovascular disease included cardiac disease, stroke and sudden death
13	Thailand Renal Replacement Therapy Registry (TRRTR)	NST	To determine the disease burden attributable to end stage renal disease, outcome, and factor influencing outcomes of renal replacement therapy	1997–2009 (n = 35,112; prevalence, new cases 7,825 in year 2009)	Longitudinal	Registry (case report)	ESRD patients from public and private dialysis centers throughout the country (440 centers)	Demographic, geographic, education, occupation, insurance scheme, income, etiology of ESRD, replacement types and termination, recipients, outcomes, death, health care and facilities, anticoagulant use, cost of treatment, number of nephrologists and surgeons, vascular access, anemia and management, cases received recombinant erythropoietin, laboratory results (Hb, Hct, ferritin, transferritin saturation, dyslipidemia laboratory results, management of renal bone diseases, nutrition status, HIV results
14	Thai Stroke Rehabilitation Registry (TSRR)	RCPT	To report the epidemiologic data and outcomes (e.g. quality of life) of the inpatient post-acute stroke rehabilitation at main tertiary hospitals in the country.	Mar–Dec 2006 (n = 327)	Before-after trial/ Longitudinal	Hospital-based registry (If the goals were reached or the BI scores were stable for 2 consecutive weeks, the program was stopped and the patients were discharged. If a patient became ill or had a serious complication that required a transfer to another department or hospital, their outcome data was not collected and the study was counted as incomplete.), Questionnaires	Stroke patients were more than 18 years old, with disability and stable medical signs for 48 h, who could follow at least 1-step commands, and who could sit without vertigo or dizziness for at least 30 min. Those with severe medical conditions, including dementia, uncontrolled heart disease, schizophrenia and multiple disabilities, were excluded.	Demographic, geographic, underlying diseases (DM, HTN, IHD, dyslipidemia), history of stroke, cerebral infraction, QOL (WHOQOL-BREF), TMSE, HADS, Barthel Index score, caregivers, cost of care, length of stay, discharge location
15	Thai National Percutaneous Coronary Intervention Registry(TPCIR)	HAT	Collect clinical data of the patients undergoing PCI in cardiac centers in Thailand.	Mar 2006 –Oct 2007 (n = 4,156)	Longitudinal	Registry	Patients who received PCI from 27 cardiac centers in Thailand	Age, sex, clinical indications for PCI, the presence or absence of heart failure, coronary risk factors (e.g. smoking), kidney disease, cerebrovascular disease, coronary artery bypass surgery, coronary anatomy, type of stent, and in-hospital outcomes (mortality, adverse events, myocardial infarction, access site complications e.g., haematoma, pseudoaneurysm, bleeding complications), cost of care
16	Thai Acute Decompensated Heart Failure Registry(Thai ADHERE)	HAT, HFCT	Collect the data of hospitalized patients with the diagnosis of heart failure (HF) or acute decompensate heart failure (ADHF) in Thailand	Mar 2006 –Nov 2007 (n = 1,612)	Longitudinal	Registry	Hospitalized patients age more than 18 years with a post discharge diagnosis of heart failure (HF) or acute decompensate heart failure (ADHF) in Thailand from 18 participating cardiac centers. Patients with cardiogenic shock, perioperative heart failure and the patients who present HF as a co-morbid condition but not a principal focus of diagnosis or treatment were excluded.	Demographic characteristics, medical history, initial evaluation, clinical presentations, hospital course, medications given prior to admission and at discharge, smoke, procedures, disposition status, discharge instruction, causes and precipitating causes of heart failure, mortality
17	Thai Parkinson’s Disease Registry (TPDR)	TRC	To gather clinical information for descriptive epidemiology and to study the accessibility and availability of Parkinson’s Disease (PD) treatment in various regions of Thailand.	Jul 2008 –Mar 2011 (n = 40,049)	Longitudinal	Registry	All individual Parkinson’s disease patients	Demographics and identifying information: name, national identification number, date of birth, gender and address; Illness-related information includes the date of diagnosis, physicians seen, symptoms and responses to medications. The sources of reporting on the patient are also recorded.
18	VigiBase	HPVC	Adverse events reports nationwide via a national network	1983 –present	Case report	Spontaneous report, Intensive report, Safety monitoring program (SMP)	All patients throughout the country.	Demographic, history of drug allergy, type of report, quality of report, causality assessment, suspected drug, adverse events
19	12-file data set	NHSO, hospitals	Administrative data for reimbursement	Oct 2009 –present (n>172,416,262 at year 2011)	Longitudinal	Routine data set record	All patients who received services at the hospitals throughout the country	**File 1**: Insurance (HN, insurance, date in and expiration date, primary and secondary hospital); **File 2**: Patient (HN, province, district, birth date, sex, marriage, occupation, nation, person identification); **File 3**: Outpatient service (HN, clinic, date of service); **File 4**: Outpatient referral (HN, date of OPD service, clinic, referral hospital, type of refer); **File 5**: Outpatient diagnosis (HN, date of diagnosis, clinic, ICD10, diagnosis type, physician); **File 6**: Outpatient operation (HN, date of OPD service, clinic, ICD9CM, physician); **File 7**: Inpatient service (HN, AN, admission date, admission time, discharge date, discharge time, discharge status, discharge type, discharge ward, department; **File 8**: Inpatient referral (AN, referral hospital, type of refer); **File 9**: Inpatient diagnosis (AN, ICD10, diagnosis type, physician); **File 10**: Inpatient operation (AN, ICD9CM, type of operation, physician, operation date start, operation time start, operation date stop, operation time stop; **File 11**: Patient charge (HN, AN, charge date, total charge, patient out-of-pocket charge, type of payment); **File 12**: Hospital finance (HN, AN, charge date, type of charge, charge of each service)
20	18-file data set	BPS, PCU	Administrative data for reimbursement, and health service	2007 –present (n>142,579,845 at year 2011)	Longitudinal	Routine data set record	All patients who received services at the PCU throughout the country	**File 1**: Person (ID, demographic, occupation, education, age, sex); **File 2**: Death (ID, death date, cause of death, in- or -out hospital death); **File 3**: Chronic disease (ID, first diagnosis, discharge); **File 4**: Card (ID, insurance, primary and secondary clinic, date issue and expiration); **File 5**: Service (ID, patient type, cost, insurance, primary and secondary clinic, payment, referral, referee clinic); **File 6**: Diagnosis (ID, date, type of diagnosis, ICD10); **File 7**: Appointment (ID, service date, appointed date, type of appointment, ICD10 for appointment); **File 8**: Surveillance (ID, ICD10, Code 506, ill date, patient status, death date, cause of death, organism); **File 9**: Drug (ID, date, drug code, price, cost, drug name, amount, unit); **File 10**: Procedure (ID, procedure code, price); **File 11**: Women (ID, type of birth control, number of children); **File 12**: Family plan (ID, type of birth control, medication, amount, clinic); **File 13**: Epidemiology (ID, date of service, type of vaccination, clinic); **File 14**: Nutrition (ID, date of service, age, weight, height, nutrition level); **File 15**: Antenatal Clinics (ID, date of service, clinic, gestational age, ANC result); **File 16**: Postpartum (baby ID, mother ID, gravida, birth date, type of birth, type of doctor, birth weight, asphyxia, Vit K, baby care, baby screening result); **File 17**: Maternal care and health (ID, gravida, last menstruation period, VDRL, HepB, HIV, Hct, Thalassemia, oral health, live born); **File 18**: Home (house ID, geographic, toilet, water, water type, garbage, house care, house durable, clean, ventilation, light, food)
	**Japan**							
1	Convergence CT Global Research Network (CGRN)	Convergence CT	Network to collect various data from medical institutions	Varies from institution to institution	Longitudinal	Receipt data, DPC data, order data, EMR data, and other clinical data from medical institutions in the Network	NA	Prescriptions (Dispensing, Date, Dosage, Duration, Unique product code), Infection, Vaccine, Procedures (Codes, Names, Date, Time), Hospitalization (admission/discharge date, medication while hospitalization, hospital discharge), Diagnosis (code: ICD-10), Lab test, Lab code, Demographics (Birth Year/Month/Date)
2	Medical Data Vision EBM Provider^®^ (EBMP)	Medical Data Vision Co.,Ltd.	Administrative database for inpatient and outpatient	From Apr 2008 (mainly from Apr,2010) (n = 4,400,000)	Longitudinal	Billing data, DPC claims, and lab Data (partial)	0–14 years old 13.5%; 15–64 year old 52.4%; 65- year old 34.1%; About 3% of Japanese population about 8.2% of total number or beds for large hospitals	Prescriptions (Date, Dosage, Duration), Unique product code, Infection, Procedures (Codes, Names, Date), Hospitalization (admission/discharge date, medication while hospitalization), Diagnosis (code: ICD-10), Lab test, Lab code, Laboratory (Basically only in Japanese but variable names are in English)
3	D*D (D star D)	Hamamatsu Med Univ.	Standardized database and a data warehouse, based on the SS-MIX scheme.	From 1999 (n = 400,000 individual patients at year 2010)	Longitudinal	EMR (Clinical database based on hospital information system in a university hospital). Medical orders including results of diagnosis tests.	0–14 years old 11%; 15–64 year old 56%; 65-year old 33%	Prescriptions (Date, Dosage, Duration, Unique product code), Infection, Procedures (Codes, Names, Date), Hospitalization (admission/discharge date, medication while hospitalization), Diagnosis (code: ICD-10), Lab test, Lab code, Demographics (Birth Year/Month/Date)
4	Osaka University (OU)	Osaka University	Clinical database within a hospital	From 1996 (n = 1,000,000)	Longitudinal	Clinical database (EMR) based on hospital information system consists of ordering, billing, and information on medical chart.	0–14 years old 13%; 15–64 year old 50%; 65- year old 36%; 100% of patients visited/hospitalized Osaka University HP	Prescriptions (Date, Dosage, Duration, Unique product code), Infection, Procedures (Codes, Names, Date), Hospitalization (admission/discharge date, medication while hospitalization, hospital discharge), Diagnosis (code: ICD-10), Lab test, Demographics (Birth Year/Month/Date)
5	Japan Medical Data Center (JMDC) Claims Database	Japan Medical Data Center	Claims database	From August 2003 (data for 320,000 subscribers is available from Jan2005) around 1,5million (based on subscribers)	Longitudinal	Claims database consists of subscribers of employees' plural Insurance programs as complete	0–14 years old 21.5%; 15–64 year old 77.3%; 65- year old 1.2%; 0.83% of Japanese population	Prescriptions (Dispensing, Dosage, Duration, Unique product code), Infection, Procedures (Codes, Names,), Hospitalization (admission/discharge date, medication while hospitalization), Diagnosis (code: ICD-10), Lab test, Lab code, Demographics (Birth Year/Month)
6	JammNet	Jamm Net CO.,LTD	Medical and dispense claims	From April 2008 (n = 600,000 from 1500 employee's Insurance programs in Japan)	Longitudinal	Medical and dispense claims from employee's insurance program	0–14 years old 20%; 15–64 year old 70%; 65 year old 10%; 1.2% of total number of claims (medical + dispense)	Prescriptions (Dispensing, Dosage, Duration, Unique product code), Infection, Procedures (Codes, Names), Hospitalization (admission/discharge date), Diagnosis (code: ICD-10), Lab code, Demographics (Birth Year/Month)
7	Medi-Trend^®^	FUJITSU FRONTECH LIMITED	Pharmacy claims database	From October 2007 (Annual number of patients: 2,600,000)	Longitudinal	Pharmacy claims database based on extramural prescription from 650 pharmacies. It contains 12.5 Million prescriptions as of Sep 2011	0–14 years old 15%; 15–64 year old 53%; 65 year old 32%	Prescriptions (Dispensing, Date, Dosage, Duration, Unique product code), Demographics (Birth Year/Month/Date)
8	IMS NPA data (IMS NPA)	IMS	Pharmacy claims database	From April 2008 (Annual number of patients: 9,700,000)	Longitudinal	Collecting extramural dispense claims from 2,500pharmacies nation-wide. 160 mil prescriptions annually	0–14 years old 14%; 15–64 year old 46%; 65 year old 40%; 6% of total extramural dispense claims	Prescriptions (Dispensing, Date, Dosage, Duration, Unique product code), Demographics (Birth Year)
9	NIHON CHOUZAI Pharmacy Claims DB (NCPCDB)	NIHON CHOUZAI Co., Ltd.	Pharmacy claims database	From April 2001 (n = 8,190,000)	Longitudinal	Claims database with extramural dispense claims and primary data (e.g. QOL) from patients visited one of the Nihon-chozai pharmacies. Nihon-chozai is the 2nd largest chain in Japan	0–14 years old 12%; 15–64 year old 54.3%; 65 year old 33.7%; 1.35% of total extramural dispense claims	Prescriptions (Dispensing, Date, Dosage, Duration, Unique product code), Demographics (Birth Year/Month/Date)
10	JMIRI Pharmacy Claims DB (JMIRI)	JMIR	Pharmacy claims database contains extramural dispense records	From January 2006 (Annual number of patients: 40,000,000)	Longitudinal	Dispense claim from pharmacies	0–14 years old 14%; 15–64 year old 54%; 65 year old 32%; 2.5% of annual prescription	Prescriptions (Dispensing, Date, Dosage, Duration, Unique product code), Demographics (Birth Year/Month)
11	RADAR	RAD-AR Council, Japan	Post marketing surveillance	Antihypertensive 1981–1996; Antihyperlipemia 1983–2006; Antihypertensive 143,509; Antihyperlipemia 32,157; oral antibacterial agent 91,797	Longitudinal	Post marketing surveillance (AE reporting) for antihypertensive & antihyperlipemia drugs provided by the RADAR Council member companies	All patients throughout the country.	Prescriptions (Date, Dosage, Duration, Unique product code), Infection, Hospitalization (medication while hospitalization), Diagnosis (code: ICD-10), Lab test, Lab code, Demographics (Birth Year/Month/Date)
12	National Health and Wellness Survey (NHWS)	Kantar Health	Survey includes more than 165 disease areas and provides a variety of metrics, such as prevalence, diagnosis and treatment rates	From 1996 (n = 25,000 at year 2008)	Cross-sectional	Primary survey research with panel members and additional patients covering US, 5 big EU, Japan, China, Brazil and Russia. NHWS collects data from patients.	0–14 years old NA%; 18–64 year old 73.7%; 65 year old 26.3%; 100% of total adult population	Prescriptions (Dispensing, Date, Dosage, Duration, Unique product code), Infection, Vaccine, Procedures (Codes, Names, Date, Time), Hospitalization (admission/discharge date, medication while hospitalization, hospital discharge), Diagnosis (code: ICD-10), Demographics (Birth Year/Month/Date, Height, Weight, Blood Pressure)
13	List of Statistical Surveys conducted by MHLW	MHLW	Survey to make fundamental materials for health policy	Every 2 years	Cross-sectional	Depending on each survey; Interview; Questionnaires; Reported materials.	Depending on each survey	There are some national surveys; Drug Price Survey, Price Survey on Special Treatment Materials, Statistics on Pharmaceutical and Medical Device Industry, Statistics of Production by Pharmaceutical Industry, Survey on Economic Conditions in Health Care(Survey on Health Care Facilities), Survey on Economic Conditions in Health Care(Survey on Health Insurers), Survey on the Insured of Employee’s Health Insurance, Survey on the Trend of Medical Care Expenditures, Estimates of National Medical Care Expenditure, etc.
14	Adverse Effects Database (AED)	PMDA	Disclosure of adverse events by law	From 2004	Case-reports	Adverse events reported by clinical institutions and companies	No study or survey population.	Suspected adverse events (cases), licensing information, year, reported by whom (profession), reported category, result, sex, age, height, weight, primary disease, suspected medicine, prescription (reason, way, amount, date), concomitant drugs, etc.
15	JapicCTI	JPIC	Information disclosure of clinical trials	Total 3077 trials.	Registry of trials	Registration	Depending on each trial.1977 trials in Japanese, 1100 trials in English, including phase 3 (525 trials) and phase 4 (42 trials). Number of Finished trials is 525.	Name of the trial, abstract, name of pharmaceuticals, name of diseases, purpose of the trials, phase, design, etc.(This is primary registry which meet criteria of WHO and The International Committee of Medical Journal Editors)
16	Rehabilitation Patients Database (JARM DB)	JARM	To make evidence for more effective and efficient rehabilitation	From 2009 (n = stroke 9,400, femoral neck fracture 3,016 at year 2011)	Longitudinal	Subject: Those who firstly received rehabilitation treatment regarding stroke, femoral neck fracture and Spinal Cord Injury. When: de-hospitalization	NA (currently Dec 2012)	Demographic data, ADL, IADL, complications
17	The Fukuoka Stroke Registry (FSR)	Kyushu University	To investigate pathophysiology and prognosis of acute stroke patients using multicenter stroke database.	From June 2007 (n = 6530 retrospective cases at July 31, 2012). Follow up data were collected in 99.7% of the patients in prospective study.	Longitudinal	Inclusion criteria: Acute stroke patients within 7 days after onset; Exclusion criteria: NA	NA	The baseline demographics and comorbidities for each patient were determined on admission. The body mass index, waist circumference, systolic and diastolic blood pressure, white blood cells, hemoglobin, total protein, low-density lipoprotein (LDL) cholesterol, high-density lipoprotein (HDL) cholesterol, triglycerides, blood glucose, hemoglobin A1c, serum creatinine (sCr), and C-reactive protein (CRP) were measured on admission.
18	The Japanese Diagnosis Procedure Combination database (JDPC)	The University of Tokyo.	To provide a standardized information platform that improves the transparency of hospital activities. Standardization, transparency and accountability.	From July 1 to October 31, 2002–2005, from July 1 to December 31, 2006–2009. Since July 2010, the survey has been conducted all year round.	Longitudinal/ Cross-sectional	All data for each patient are recorded at discharge. The hospitals send all anonymous data for each month to the DPC research group, and the data are compiled in the database server in the Department of Health Management and Policy, The University of Tokyo.	NA on details; The number of patients included in 2009 (2.57 million) represented approximately 40% of all the inpatient admissions to acute care hospitals in Japan.	Hospital information; Patient background information; Diagnoses; Procedures; Admission and discharge data; Claim data; Detailed clinical data; Global Assessment of Functioning (GAF) scale
19	Database of Medical Device (Mdevice)	MEDIS-DC	Catalogue of medical devices. Reimbursed prices are linked to each medical device. Total reimbursed price are automatically calculated.	From 2005.	Registry of devices	Registered by R&D company	NA	Name of devices, MRP, unit of reimbursement, reimbursement price, medical affair code, total reimbursement price of combination device, day of revised reimbursement price, class names of categories
20	Japanese Study of Aging and Retirement (JSTAR)	RIETI	Panel data to reveal needs for social security, including economic, social and health variables.	2007 first wave; 2009 second wave; 2011 third wave; (n = 16,000 at year 2011)	Longitudinal	Self-completion or "drop off" questionnaire, computer-assisted personal interview. Subjects are randomly selected based on resident registry.	50–75 years old	Include diverse information on the economic, social, and health conditions of elderly people. In addition, the survey is designed to ensure, to the maximum extent possible, comparability with preceding surveys such as the Health and Retirement Study (HRS) in the United States, the Survey of Health, Aging and Retirement in Europe (SHARE) in continental Europe, and the English Longitudinal Study of Aging (ELSA) in the United Kingdom.

***Note*: AED**, Adverse Effects Database; **BMI**, Body Mass Index; **BN**, Bureau of Nutrition Department of Health; **BNCD**, Bureau of Non-Communicable Disease; **BP**, Blood Pressure; **BPS**, Bureau of Planning and Strategy; **BRFSS**, Behavioral Risk Factors Surveillance System; **CA,** Cancer; **CBC**, Complete Blood count; **CGRN**, ConvergenceCT Global Research Network; **COPD**, Chronic Obstructive Pulmonary Disease; **CR**, Cancer Registry; **DM**, Diabetes Mellitus; **DMH**, Department of Mental Health; **EBMP**, Medical Data Vision EBM Provider^®^;**EKG,** Electrocardiography; **ESRD**, End-Stage Renal Disease; **FBS**, Fasting Blood Sugar; **FSR**, The Fukuoka Stroke Registry; **HAT**, The Heart Association of Thailand under the Royal Patronage; **Hb**, Hemoglobin;**HbA1c**, Hemoglobin A1c;**Hct**, Hematocrit; **HDL**, High-density Lipoprotein; **HFCT**, Heart Failure Council of Thailand; **HIV/AIDS**, Human Immunodeficiency Virus/Acquired Immunodeficiency Syndrome; **HPVC**, Health Product vigilance Center Thai FDA;**HSRI**, Health System Research Institute; **HTN**, Hypertension; **HWS**, Health and Welfare Survey; **IHD**, Ischemic Heart Disease; **IMS NPA**, IMS NPA data; **JARM DB**, Rehabilitation Patients Database; **JARM**, the Japanese Association of Rehabilitation Medicine; **JDPC**, The Japanese Diagnosis Procedure Combination database; **JMDC**, Japan Medical Data Center Claims Database; **JMIRI**, JMIRI Pharmacy Claims DB; **JPIC**, Japan Pharmaceutical Information Center; **JSTAR**, Japanese Study of Aging and Retirement; **LDL**, Low-density Lipoprotein; **Mdevice**, Database of Medical Device; **MEDIS-DC**, Medical Information System Development Center; **MHLW**, List of Statistical Surveys conducted by MHLW;**MHLW**, Ministry of Health, Labor and Welfare; **MICS**, Multiple Indicator Cluster Survey; **NCI**, National Cancer Institute; **NCPCDB**, NIHON CHOUZAI Pharmacy Claims DB; **NDS**, National Disability Survey; **NESMH**, National Epidemiology Survey on Mental Health; **NHES**, National Health Examination Survey; **NHSO**, National Health Security Office; **NHWS**, National Health and Wellness Survey; **NNS**, National Nutrition Survey; **NSO**, National Statistical Office; **NST**, Nephrology Society of Thailand; **OU**, Osaka University; **PCI**, Percutaneous Coronary Intervention; **PCU**, Primary care unit; **PHC**, Population and Housing Census; **PMDA**, Pharmaceutical and Medical Devices Agency; **QOL**, Quality of Life; **RAD-AR**, Risk/benefit Assessment of Drug-Analysis & Response; **RCPT**, The Royal College of Physiatrists of Thailand; **RHS**, Reproductive Health Survey; **RIETI**, The Research Institute of Economy, Trade and Industry; **SES**, Socio-Economic Survey; **TC**, Total Cholesterol; **TDR**, Thai Diabetes Registry; **TES**, Thailand Endocrinology Society; **TG**, Triglyceride; **Thai ADHERE**, Thai Acute Decompensated Heart Failure Registry; **TPCIR**, Thai National Percutaneous Coronary Intervention Registry; **TPDR**, Thai Parkinson’s Disease Registry; **TRC**, Thai Red Cross Society; **TRRTR**, Thailand Renal Replacement Therapy Registry; **TSRR**, Thai Stroke Rehabilitation Registry

**Table 3:** Databases potential for health technology assessment research.*

Domain Outcomes/variables	Thailand																				Japan																			
	1:PHC	1:HWS	1: SES	1: RHS	1: NDS	1: MICS	1: NHES	1: NESMH	1: NNS	2: BRFSS	2: CR	2: TDR	2: TRRTR	2: TSRR	2: TPCIR	2: Thai ADHERE	2: TPDR	2: VigiBase	3: 12-File	3: 18-File	1: NHWS	1: MHLW	1: JSTAR	2: RADAR	2: AED	2: JapicCTI	2: RPD	2: FSR	3: CGRN	3: EBMP	3: D star D	3: OU	3: JMDC	3: JammNet	3: Medi-trend^®^	3: IMS NPA	3: NCPCDB	3: JMIRI	3: JDPC	3: Mdevice
**Safety/Efficacy/ Effectiveness**																																								
Mortality	•			•							•	•	•		•	•		•	•	•		•			•	•		•	•		•	•							•	
Morbidity		•			•	•	•	•	•	•	•	•	•	•	•	•		•	•	•	•	•		•	•	•	•	•	•	•	•	•	•						•	
Disability					•	•	•																•		•	•	•				•	•							•	
Adverse event														•	•			•	•	•				•	•	•					•	•							•	
Quality of life														•									•																	
Life year, QALY														•																										
**Social/ethical**																																								
Compliance																					•																			
Acceptance																							•																	
Satisfaction																					•		•																	
**Organizational/ professional**																																								
Utilization (e.g. service or technology utilization)	•	•	•	•	•	•	•			•	•	•	•	•	•	•	•		•	•	•	•							•	•	•	•	•	•	•	•	•	•	•	
Length of stay														•		•			•		•	•							•	•	•	•							•	
Personnel required						•																•																		
Material required (e.g. hospital bed)																						•																	•	
**Economic**																																								
Cost/price		•	•						•				•	•	•				•	•		•																	•	•
Income/economic status	•	•	•				•	•	•	•			•									•	•																	
**Epidemiological**																																								
Prevalence		•		•	•	•	•	•	•	•	•	•	•	•	•	•	•		•	•	•	•	•				•	•	•		•	•							•	
Incidence				•			•	•	•	•	•	•	•	•	•	•	•	•	•	•	•	•		•		•	•	•	•	•	•	•							•	
Health state		•		•	•	•	•	•	•	•	•	•	•	•	•	•	•	•	•	•	•	•	•	•	•	•	•	•	•	•	•	•							•	
Demographic (e.g. age, sex, education)	•	•	•	•	•	•	•	•	•	•	•	•	•	•	•	•	•	•	•	•	•	•	•	•	•	•	•	•	•	•	•	•	•	•	•	•	•	•	•	

***Note*:**

* Number in front of each database indicated type of database (1 = population, household and health survey, 2 = disease surveillance and registries, 3 = administrative and claimed database).

**AED**, Adverse Effects Database; **BRFSS**, Behavioral Risk Factors Surveillance System; **CGRN**, Convergence CT Global Research Network; **CR**, Cancer Registry; **EBMP**, Medical Data Vision EBM Provider^®^;**FSR**, The Fukuoka Stroke Registry; **HWS**, Health and Welfare Survey; **IMS NPA**, IMS NPA data; **JARM DB**, Rehabilitation Patients Database; **JDPC**, The Japanese Diagnosis Procedure Combination database; **JMDC**, Japan Medical Data Center Claims Database; **JMIRI**, JMIRI Pharmacy Claims DB;**JSTAR**, Japanese Study of Aging and Retirement; **Mdevice**, Database of Medical Device; **MHLW**, List of Statistical Surveys conducted by MHLW;**MICS**, Multiple Indicator Cluster Survey; **NCPCDB**, NIHON CHOUZAI Pharmacy Claims DB; **NDS**, National Disability Survey; **NESMH**, National Epidemiology Survey on Mental Health; **NHES**, National Health Examination Survey; **NHWS**, National Health and Wellness Survey; **NNS**, National Nutrition Survey; **OU**, Osaka University; **PHC**, Population and Housing Census; **RHS**, Reproductive Health Survey; **SES**, Socio-Economic Survey; **TDR**, Thai Diabetes Registry; **Thai ADHERE**, Thai Acute Decompensated Heart Failure Registry; **TPCIR**, Thai National Percutaneous Coronary Intervention Registry; **TPDR**, Thai Parkinson’s Disease Registry; **TRRTR**, Thailand Renal Replacement Therapy Registry; **TSRR**, Thai Stroke Rehabilitation Registry

Out of 20 databases from Thailand, all databases (100%) contained national data. Fourteen databases (70%) comprised of more than 10,000 individuals, 15 databases (75%) provided information on chronic diseases, and 9 databases (45%) had longitudinal data. After classified into three groups according to sources and method of data collection, the first group contained 9 databases of surveys of selective samples of the populations or households. These contained information on population health status (e.g. mortality, and morbidity), health states, and behaviors of specific interests, for examples, mental health, risk factors, physical exercise, and nutrition. The second group contained 9 databases of disease surveillances and registries. In this group, two surveillance databases were primarily population-based, while the other seven registry databases were restricted to patients who had visited health care facilities, but were not population-based. There was a lack of economic information in facility-based data. The third group contained 2 databases obtained from hospital-based administrative data for reimbursement. Cost/payment for health care, utilization of services, morbidity, and mortality were available in electronic databases (Table 3).

Out of 20 databases from Japan, 17 databases (85%) represented national level data. Thirteen databases (65%) consisted of more than 10,000 individuals, 8 databases (40%) have information on chronic diseases, and 15 databases (75%) provided longitudinal data. The first group comprised of 3 databases. Some included social aspects (e.g. patients’ satisfaction) and economic variables as well as population health status. The second group contained 5 databases obtained from disease surveillances and registries. In this group, we obtained population health status (e.g. mortality, mobility, prevalence) but without economic variables. The third group contained 12 databases obtained from hospital-based administrative data for reimbursement or claimed data. We included private services on claimed data in this group and regarded claimed data as utilization information. It is noteworthy that we have information on reimbursement price of each medical device in Databases of Medical Device which are freely accessible (Table 3).

**Fig 1:**
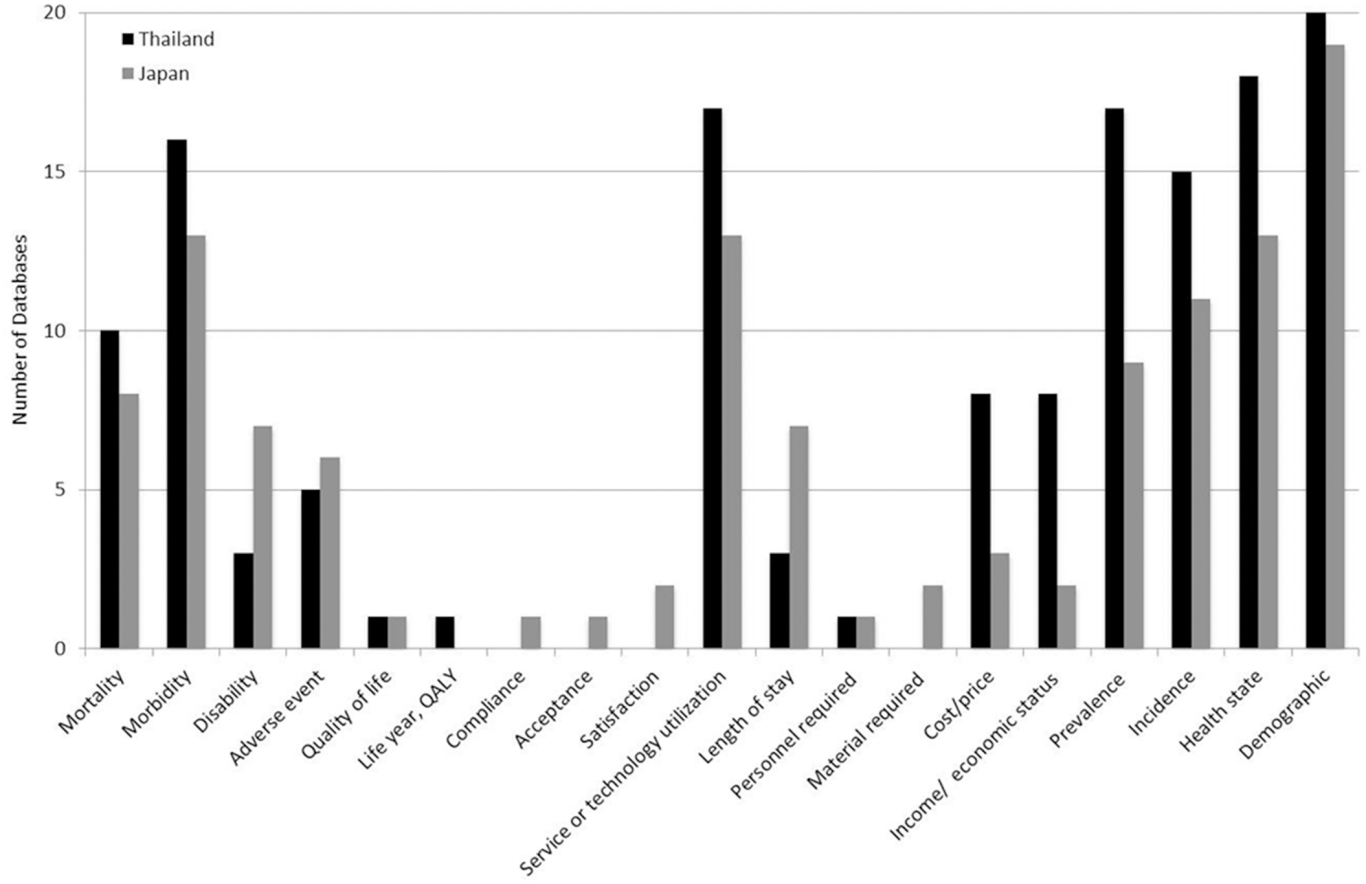
Number of databases in each domain.

These data have potential for use in HTA research, according to the criteria in Methods section. All databases from Thailand (100%) provided epidemiological data such as incidence, prevalence, and health state, but 19 of 20 databases (95%) from Japan provided such information. Data on safety/efficacy/effectiveness (e.g. mortality, morbidity, disability, adverse events, quality of life) were found in 18 of 20 databases (90%) from Thailand, and 14 of 20 (70%) from Japan. While 2 databases (10%) from Japan provided information on the social/ethical issues, which were not found in the databases from Thailand. For organizational/professional information (e.g. service/technology utilization, length of stay, personnel required, material required), 17 (85%) and 13 (65%) of 20 databases from Thailand and Japan respectively, provided such information. In addition, data on economics were also found in 12 (60%) and 4 (20%) of 20 databases from Thailand and Japan respectively (Table 3). Thus, databases which contained information about disability, adverse event, quality of life, social/ethical issues, and organization/professional issues were limited (Table 3 and Fig 1). Nevertheless, access to patient-level data was also limited since some databases were not available on the websites. In addition some were only available as in-house service or as commercial use (S1 Table). To obtain data at patient level, formal contact with database’s host was needed.

## Discussion

To the best of our knowledge, this is the first study in Asia-Pacific region that provides the list of healthcare databases and assessed their potential for HTA research. In the present study, a sample of 40 large-scale databases (20 from Thailand and 20 from Japan) implies an abundance of data with high potential for HTA research.

Although, most people may not expect that a number of healthcare databases are available in Thailand, the adoption of universal coverage and the use of case-mix classification system partly render the development of electronic claims database in early 2000. This is consistent with Japan. It is possible to question whether having this compilation of databases available to the public would make a difference in terms of accessibility. It has been shown in a number of reports that a lack of listing of databases can be one of the major causes of minimal use of existing databases, having a list of databases demonstrates the availability of databases.

Using databases for HTA research particularly for coverage decision is not uncommon [17]. These databases generally contained large sample of subjects and were designed with appropriate sampling to be representative of national level. Most importantly, database analysis can be performed with minimal time and cost. Database-based study, for example adverse event surveillance reports [18], is useful for rare disease/events that sometimes are never reported in randomized controlled trials (RCTs). Databases that were collated from routine services provide ‘real-world’ data that are essential for HTA research. Furthermore, databases have multiple functions for applications. For example, Health and Welfare Survey (HWS) database provides the information on morbidity, service or technology utilization, cost/price, prevalence, health state, and demographic data, which can be used in various purposes such as cost estimation, disease incidence/prevalence, and outcomes ascertainment.

Database-based studies have both strengths and limitations. For all non-randomized data, the most significant concern is the potential for bias. Database studies do not meet the methodological rigor of RCTs, regardless of the use of statistical approaches to adjust for selection bias [19,20]. Thus, database-based studies need a rigorous identification the sources of bias and confounding, and then adjusted for these before estimating the impact of interventions. In addition, routine databases usually encounter with the problems of collection errors and missing data. Subsequently, justification of data selection or data imputation should be clarified before estimating the impact of interventions or cost estimation as well. However, the different types of data sources have their own characteristics and limitations.

Population, household and health surveys are designed to collect data of the population (e.g. household, economic status, health status and well-being, healthcare utilization and expenditure, and treatment patterns). They typically collect information from individuals in target population using rigor methodologies i.e. relying on proper sample designs. With these methods, surveys can provide information on the target population. Accordingly, health surveys data can make unique contributions in terms of generalizability of treatments and their impacts, and utilization of health services. The major limitation of surveys for HTA research is the lack of data on specific products. In addition, the issues of subjectivity and recall bias should always be considered [21].

Disease surveillances are reports of patients who have experiences with diseases or unexpected events, while registries are prospective studies of patients who have a particular disease and/or receiving a particular intervention. They can be used for understanding natural history, assessing and monitoring safety and effectiveness, evaluating quality of care and performance, and estimating cost-effectiveness [22]. They include larger and more diverse group of patients than that in RCTs. Thus, they reflect real world patients, practices, and outcomes. Moreover, patients are always followed over a long time frame, allowing for long-term outcomes assessment. Treatment patterns are reflecting the everyday clinical decision-making that is most relevant to providers, decision makers, and payers as well. Since disease surveillances and registries intended to capture data on a population of interest, the inherent limitations of this kind of observational studies should be considered. This kind of database may not be suitable for hypothesis testing, but are useful for hypothesis generation. In addition, incidence and prevalence derived from disease surveillances and registries may not be accurate because they always came from case registers, thus lacking actual denominator/population. However, they remain useful for the estimation of burden of disease.

Administrative and claims databases, in general, are collected primarily for reimbursement purposes, but contained some clinical variables; procedure used, and charge information. Claims databases usually lend to longitudinal and cross-sectional data collection of clinical and economic outcomes at patient, group, or population level. Thereby, they can be used for HTA research at low cost and in a short time. Given the large data size of administrative and claims databases, rare events/outcomes could be potentially detected. In addition, the databases also demonstrate their usefulness for measuring economic impact and association between interventions and outcomes [23,24]. The use of this kind of databases had been challenged due to a number of factors including: 1) data quality e.g. missing data, and coding errors, 2) limited information on clinical outcomes and symptoms, 3) absence of population denominator, 4) lack of information of the difference between cost and charge. However, the large data size may overcome the issue of random missing data. Sometimes the estimation of treatment effects and costs can be biased due to a correlation between unobserved variables that are associated with treatment selection and outcomes, i.e. baseline characteristics.

In Thailand, Medical Research Foundation is developing a website to collect databases/registries [25], and Ganesh SAP Research Unit is collecting the published outcomes research and health economics studies, reports, theses, and proceedings which were conducted in Thailand [26,27]. In Japan there is also a development of a single computerized database linking medical records from all hospitals in Japan [28]. These make it increasingly possible for rapid reviews that can be used for HTA and provide the information for decision making.

Another important aspect that should be considered while using databases is the need to balance data access with need to protect patient confidentiality. To protect the rights and interests of patients, it is important to ensure that all possible steps are undertaken to limit access to confidential information. To minimize risks of disclosure, informed consent for the use of clinical data should be obtained if possible [29] The most effective strategy to keep confidentiality is to remove all identifying information from medical records before any use. [29] However, in some situations, patient identifying information is needed. For example, researcher needs patient identification to link information across sources (e.g. death records, pharmacy data, hospitalization data). Idealistically, informed consent should be obtained from each patient. In reality, samples used in analysis based on computerized database often includes more than thousands of cases, causing acquisition of individual informed consent impossible. Thus, investigators have to demonstrate a clear plan to adequately protect identifiers and prevent disclosure of confidential information. In addition, identifying information should be destroyed at the earliest opportunity.

The main limitation of the present study was that we did not perform a comprehensive search of all existing healthcare databases in both Thailand and Japan. However, we try our best by including relevant databases to describe/illustrate the characteristics and potential use for HTA to the readers/researchers.

In summary, our study provides data sources where HTA researchers can use as an initial source for data search. It should be noted that the use of databases for HTA research has strengths and limitations. The readers/decision makers are referred to comprehensive summaries and critical appraisals [30] about strengths and limitations noted in the literature before using database-based studies for HTA. Further study describing how databases have been used in HTA is warranted.

## Supporting Information

S1 Table Database request route. (PDF)
