# Supplementary material for: Healthcare Databases in Thailand and Japan: Potential Sources for Health Technology Assessment Research
Source: PLoS One. 2015 Nov 11;10(11):e0141993. doi: 10.1371/journal.pone.0141993 (PMC4641604; doi:10.1371/journal.pone.0141993)
Supplement: S1 Table — (PDF) [file pone.0141993.s001.pdf]

Supplement material

S1 Table Database request route

	Database	Agent	Website/email	Remark
**Thailand**				
1	Population and Housing Census(PHC)	National Statistical Office (NSO) Statistical Information Service and Dissemination Group, Statistical Forecasting Bureau, The Government Complex, Building B, ChaengWatthana Rd, Laksi Bangkok 10210 Thailand Tel: (66) 21417500-03, Fax: (66) 21438132	http://www.nso.go.th, Email: services@nso.go.th	Public service Individual data: directly contact needed Aggregate data: freely retrieved via internet
2	Health and Welfare Survey (HWS)	NSO (same as above)	same as above	same as above
3	Socio-Economic Survey (SES)	NSO (same as above)	same as above	same as above
4	Reproductive Health Survey (RHS)	NSO (same as above)	same as above	same as above
5	National Disability Survey (NDS)	NSO (same as above)	same as above	same as above
6	Multiple Indicator Cluster Survey(MICS)	NSO (same as above)	same as above	same as above
7	National Health Examination Survey(NHES)	Health System Research Institute (HSRI) 4th Floor, National Health Building, 88/39 Tiwanon 14 Road, Muang District, Nonthaburi 11000Thailand Tel:(66) 28329200	http://www.hsri.or.th, Email: hsri@hsri.or.th	Public service Individual data: directly contact needed Aggregate data: freely retrieved via internet
8	National Epidemiology Survey on Mental Health (NESMH)	Department of Mental Health (DMH) Ministry of Public Health 88/20 Moo 4 Tiwanon 14 Road, Muang District,Nonthaburi 11000Thailand Tel: (66) 21495555-60	http://www.dmh.go.th	Public service Individual data: directly contact needed Aggregate data: freely retrieved via internet
9	National Nutrition Survey (NNS)	Bureau of Nutrition (BN) Department of Health, Ministry of Public Health, Muang District,Nonthaburi 11000Thailand Tel: (66) 25904328 Fax: (66) 25904339	http://nutrition.anamai.moph.go.th	Public service Individual data: directly contact needed Aggregate data: freely retrieved via internet
10	Behavioral Risk Factors Surveillance System(BRFSS)	Bureau of Non-Communicable Disease (BNCD), Ministry of Public Health, 3rd Floor, Boromarajonani College of Nursing Bamrasnaradura, Tiwanon Road, Muang District,Nonthaburi 11000Thailand Tel: (66) 25903987 Fax: (66) 29510273	http://thaincd.com	Public service Individual data: directly contact needed Aggregate data: freely retrieved via internet
11	Cancer Registry (CR)	National Cancer Institute (NCI) 268/1 Rama VI Ratchathewi Bangkok 10400 Tel: (66) 23547025, (66) 23547028-35 Fax: (66) 23547037	http://www.nci.go.th E-mail: admin@ncicheckup.com	Public service Individual data: directly contact needed Aggregate data: freely retrieved via internet
12	Thai Diabetes Registry(TDR)	Thailand Endocrinology Society (TES) 10th Floor, Royal Golden Jubilee Building, Soonvijai, New Petchburi Road, Bangkok 10310 Thailand Tel: (66) 27166661-4 ext 1041 Fax: (66) 27166661-2 ext 1042	http://www.thaiendocrine.org	Public service Individual data: directly contact needed Aggregate data: freely retrieved via internet
13	Thailand Renal Replacement Therapy Registry (TRRTR)	Nephrology Society of Thailand (NST) 4th Floor, Royal Golden Jubilee Building, Soonvijai, New Petchburi Road, Bangkok 10310 Thailand Tel: (66) 27166091, (66) 27167450 Fax: (66) 27181900	http://www.nephrothai.org E-mail: sathit@edu.vajira.ac.th	Public service Individual data: directly contact needed Aggregate data: directly contact needed
14	Thai Stroke Rehabilitation Registry(TSRR)	The Royal College of Physiatrists of Thailand (RCPT), 10th Floor, Royal Golden Jubilee Building, Soonvijai, New Petchburi Road, Bangkok 10310 Thailand Tel: (66) 27166808, Fax: (66) 2716809	http://www.rehabmed.or.th/royal/rc_thai	Public service Individual data: directly contact needed Aggregate data: directly contact needed
15	Thai National Percutaneous Coronary Intervention Registry(TPCIR)	The Heart Association of Thailand under the Royal Patronage (HAT), 5th Floor, Golden Jubilee Building, Soonvijai, New Petchburi Road, Bangkok 10310 Thailand Tel: (66) 7180060-5, Fax: (66) 27180065	http://www.thaiheart.org Email: thaiheart@thaiheart.org	Public service Individual data: directly contact needed Aggregate data: directly contact needed
16	Thai Acute	The Heart Association of Thailand under	http://www.thaiheart.org	Public service

	Decompensated Heart Failure Registry(Thai ADHERE)	the Royal Patronage (HAT), 5th Floor, Golden Jubilee Building, Soonvijai, New Petchburi Road, Bangkok 10310 Thailand<br>Tel: (66) 7180060-5, Fax: (66) 27180065	Email: thaiheart@thaiheart.org	Individual data: directly contact needed<br>Aggregate data: directly contact needed
17	Thai Parkinson's Disease Registry (TPDR)	Thai Red Cross Society (TRC)<br>1871 Terdprakiat Bld., Henry Dunant Rd., Patumwan, Bangkok 10330 Thailand	http://www.redcross.or.th<br>Email: intertrc@redcross.or.th	Public service<br>Individual data: directly contact needed<br>Aggregate data: directly contact needed
18	VigiBase	Health Product vigilance Center (HPVC)<br>Food and Drug Administration<br>Ministry of Public Health<br>88/24 Moo 4 Tiwanon 14 Road, Muang District,Nonthaburi 11000Thailand<br>Tel: (66) 25907307	http://thaihpvc.fda.moph.go.th<br>Email: adr@fda.moph.go.th	Public service<br>Individual data: directly contact needed<br>Aggregate data: directly contact needed
19	12-file data set	National Health Security Office (NHSO)<br>The Government Complex, Building B, ChaengWatthana Rd, Laksi Bangkok 10210 Thailand<br>Tel: (66) 21414000, Fax: (66) 21439730<br>OR<br>All hospitals in Thailand	http://www.nhso.go.th	Public service<br>Individual data: directly contact needed<br>Aggregate data: directly contact needed
20	18-file data set	Bureau of Planning and Strategy (BPS)<br>Ministry of Public Health<br>TiwanonRoad, Muang District,Nonthaburi 11000Thailand<br>Tel: (66) 25901504<br>OR<br>All Primary Care Units (PCU) in Thailand	http://bps.ops.moph.go.th	Public service<br>Individual data: directly contact needed<br>Aggregate data: freely retrieved via internet
**Japan**				
1	ConvergenceCT Global Research Network (CGRN)	Yokohama Sky building 20F<br>2-19-12 Takashima,Nishi Ward, Yokohama, Kanagawa Prefecture, 200-0011 Japan<br><br>TEL: 045-440-6570<br>FAX: 045-440-6001<br>Email: info@convergencect.com	http://www.convergencect.com/<br>http://www.convergencect.com/japan/	Commercial service<br>Individual data: directly contact needed<br>Aggregate data: directly contact needed<br><br>Access to raw data via web: limited<br>Access to original medical data: limited<br>Data update: monthly
2	Medical Data Vision EBM Provider® (EBMP)	Medical Data Vision Co.,Ltd.<br>7 Kanda Mitoshirochō, Chiyoda-ku, Tokyo, 101-0053 Japan	http://www.mdv.co.jp/	Commercial service<br>Individual data: directly contact needed<br>Aggregate data: directly contact needed<br><br>Access to raw data via web: limited<br>Access to original medical data: No<br>Data update: monthly
3	D star D	Department of Medical Informatics, Hamamatsu Medical University, Japan<br>1-20-1 Handayama, Hamamatsu-city,Shizuoka 431-3192 Japan<br>Tel: +81-53-435-2111<br>Fax: +81-53-435-2112	http://www.hama-med.ac.jp/	In-the-organization service<br>Individual data: intranet only<br>Aggregate data: intranet only<br><br>Access to raw data via web: limited (intranet only)<br>Access to original medical data: Yes<br>Data update: real-time
4	Osaka University	Yasushi Matsumura, MD, PhD, Department of Medical Information Science,Osaka University Hospital, 2-15 Yamada-oka, Suita, Osaka, 565-0871 Japan.Tel: 81-6-879-5900 Fax: 81-6-879-5903 E-mail:secretary@hpinfo.med.osaka-u.ac.jp<br>matumura@hp-info.med.osaka-u.ac.jp	http://www.hosp.med.osaka-u.ac.jp/home/hp-info/en/index.html	In-the-organization service<br>Individual data: directly contact needed<br>Aggregate data: directly contact needed<br><br>Access to raw data via web: No<br>Access to original medical data: Yes<br>Data update: every day
5	Japan Medical Data Center (JMDC) Claims Database	Kojimachi 311 Building 2F, Kojimachi 3-1, Chiyoda-ku, Tokyo 102-0083<br>Tel: (81) 3-3511-6780 (main)<br>Mail:info@jmdc.co.jp	http://www.jmdc.co.jp/en/index.html	Commercial service<br>Individual data: directly contact needed<br>Aggregate data: directly contact needed<br><br>Access to raw data via web: Yes<br>Access to original medical data: No<br>Data update: monthly

6	JammNet	Shibadaimon, Minato-ku, Tokyo, 105-0012 Japan<br>TEL (03)5401-0311	http://www.jamm-net.co.jp/	Commercial service<br>Individual data: directly contact needed<br>Aggregate data: directly contact needed<br><br>Access to raw data via web: limited<br>Access to original medical data: No<br>Data update: monthly
7	Medi-Trend®	2-6-5 Nihonbashikayabachō, Chūō-ku, Tokyo, 103-0025 Japan<br>Tel: 03-6661-0892<br>Fax: 03-6661-1763	http://www.mfc-net.com/	Commercial service<br>Individual data: directly contact needed<br>Aggregate data: directly contact needed<br><br>Access to raw data via web: Yes<br>Access to original medical data: No<br>Data update: monthly
8	IMS NPA data (IMS NPA)	4-1-28Toranomon Minato, Tokyo 105-0001 Japan	http://www.imshealth.com/portal/site/ims	Commercial service<br>Individual data: directly contact needed<br>Aggregate data: directly contact needed<br><br>Access to raw data via web: limited<br>Access to original medical data: No<br>Data update: monthly
9	NIHON CHOUZAI Pharmacy Claims DB (NCPCDB)	NIHON CHOUZAI Co.,Ltd.<br>1-9-1 Marunouchi, Chiyoda, Tokyo 100-6737	http://www.nicho.co.jp/	Commercial service<br>Individual data: directly contact needed<br>Aggregate data: directly contact needed<br><br>Access to raw data via web: limited<br>Access to original medical data: No<br>Data update: real-time
10	JMIRI Pharmacy Claims DB (JMIRI)	5-24-2 Hongo, Bunkyo-ku, Tokyo, 113-0033 Japan	http://www.jmiri.jp/index.php	Commercial service<br>Individual data: directly contact needed<br>Aggregate data: directly contact needed<br><br>Access to raw data via web: limited<br>Access to original medical data: No<br>Data update: monthly
11	RADAR	1-4-2 Nihonbashihoridomechō, Chuo-ku, Tokyo, 103-0012<br>TEL :03(3663)8891<br>FAX :03(3663)8895<br>e-mail info@ad-ar.or.jp	http://www.rad-ar.or.jp/	Public service<br>Individual data: directly contact needed<br>Aggregate data: directly contact needed<br><br>Access to raw data via web: limited<br>Access to original medical data: No<br>Data update: monthly
12	National Health and Wellness Survey (NHWS)	For more information on the National Health and Wellness Survey, please contact nhws@kantarhealth.com.	http://www.kantarhealth.com/	Commercial service<br>Individual data: directly contact needed<br>Aggregate data: directly contact needed<br><br>Access to raw data via web: No<br>Access to original medical data: No<br>Data update: every year
13	List of Statistical Surveys conducted by Ministry of Health, Labor and Welfare (MHLW)	Statistics and Information Department Minister's Secretariat<br>Ministry of Health, Labour and Welfare<br>1-2-2 KasumigasekiCihyoda-ku Tokyo, 100-8916 Japan<br>Tel :+81-(0)3-5253-1111<br>www-admin@mhlw.go.jp	http://www.mhlw.go.jp/english/database/index.html	Public service<br>Individual data: directly contact needed<br>Aggregate data: freely retrieved via internet
14	Adverse Effects Database (AED)	3-3-2 Kasumigaseki, Chiyoda-ku, Tokyo, 100-0013 Japan<br><br>PMDA<br>03-3506-9003	http://www.info.pmda.go.jp/fsearchnew/jsp/menu_fukusayou_base.jsp	Public service<br>Individual data: freely retrieved via internet<br>Aggregate data: freely retrieved via internet

		fukusayou-database@pmda.go.jp		
15	Clinical Trials Information (JapicCTI)	2-12-15 Shibuya, Shibuya-ku, Tokyo, 150-0002 Japan<br>TEL: 03-5466-1811	http://www.clinicaltrials.jp/user/ctiMenu.jsp<br>http://www.japic.or.jp/	Public service<br>Individual data: directly contact needed<br>Aggregate data: freely retrieved via internet
16	Rehabilitation Patients Database (JARM DB)	6-32-3, Kagurazaka, Shinjuku-ku, Tokyo, 162-0825 Japan<br>The Japan Association of Rehabilitation Medicine (JARM)<br>E-mail:office@jarm.or.jp<br>JARD office: rehadb-admin@umin.org	http://square.umin.ac.jp/JARD/index.html<br>http://square.umin.ac.jp/jarm-db/index.html	Public service<br>Individual data: limited to member of the association or contributors<br>Aggregate data: limited to member of the association or contributors
17	The Fukuoka Stroke Registry (FukuokaSR)	Fukuoka Stroke Registry<br>Department of Clinical Science, Graduate School of Medical Sciences, Kyushu University<br>3-1-1 Maidashi, Higashi-ku, Fukuoka 812-8582 JAPAN<br>TEL +81-92-642-5256<br>kamouchi@intmed2.med.kyushu-u.ac.jp	http://www.fukuoka-stroke.net/english/index.html	Public service<br>Individual data: directly contact needed<br>Aggregate data: limited
18	The Japanese Diagnosis Procedure Combination database (JDPC)	Department of Health Management and Policy<br>Graduate School of Medicine, The University of Tokyo<br>7-3-1 Bunkyo-ku, Hongo, Tokyo 1138655, Japan<br>Phone: +81-3-5800-9158<br>FAX: +81-3-5800-9164<br>E-mail: hmp@umin.ac.jp	http://plaza.umin.ac.jp/~hmp/cgi-bin/wiki/wiki.cgi?page=Department+of+Health+Management+and+Policy	Public service<br>Individual data: directly contact needed<br>Aggregate data: directly contact needed
19	Database of Medical Device (Mdevice)	1-1, Kagurazaka, Shinjuku-ku, Tokyo, 162-0825 Japan<br>Mail ：kikidb@medis.or.jp<br>TEL ：03-3267-1924<br>FAX ：03-3267-1931	https://www.kikidb.jp/<br>http://search.kikidb.jp/	Public service<br>Individual data: freely retrieved via internet<br>Aggregate data: freely retrieved via internet
20	Japanese Study of Aging and Retirement (JSTAR)	Research Institute of Economy, Trade and Industry (Attn: JSTAR)<br>11th floor, Annex, Ministry of Economy, Trade and Industry (METI)<br>1-3-1, Kasumigaseki, Chiyoda-ku<br>Tokyo, Japan 100-8901	http://www.rieti.go.jp/en/projects/jstar/index.html	Public service<br>Individual data: directly contact needed<br>Aggregate data: directly contact needed
